# Long noncoding RNA HOTAIR facilitates pulmonary vascular endothelial cell apoptosis via DNMT1 mediated hypermethylation of Bcl-2 promoter in COPD

**DOI:** 10.1186/s12931-022-02234-z

**Published:** 2022-12-17

**Authors:** Zhongshang Dai, Xiangming Liu, Huihui Zeng, Yan Chen

**Affiliations:** Second Xiangya Hospital of Central South University, Changsha, China

## Abstract

**Background:**

To study the regulatory effect of Long non-coding RNA (LncRNA) HOX transcript antisense RNA (HOTAIR) on pulmonary vascular endothelial cell (HPVEC) apoptosis and determine whether the HOTAIR facilitate HPVEC apoptosis via DNMT1 mediated hypermethylation of Bcl-2 promoter in chronic obstructive pulmonary disease (COPD).

**Methods:**

LncRNA array was used to measure the differentially expressed lncRNAs in COPD and non-COPD lung tissues. Expression of HOTAIR in COPD patient lungs and cigarette smoke extract (CSE)-induced HPVEC was assessed by qRT-PCR. The location of HOTAIR was determined in COPD patient lungs and HPVEC by RNA in situ hybridization (RNA-ISH). The emphysema mouse model and HOTAIR knockdown mice were each established by inhaling cigarette smoke or intratracheal lentiviral vectors instillation. The dysregulation of DNA methyltransferase enzyme 1 (DNMT1), B-cell lymphoma-2 (Bcl-2), Bcl-2-associated X protein (Bax) and Cleaved-caspase 3 protein expression were detected by Western blotting. HOTAIR, DNMT1, Bcl-2 and Bax mRNA expression were measured by quantitative real-time polymerase chain reaction. TUNEL (terminal deoxynucleotidyl transferase dUTP nick end labeling) assays were used to assess apoptotic ratio in mice and CSE-induced HPVEC. Methylation-specific PCR (MSP) assay was conducted to observe the alterations in the methylation of the Bcl-2 promoter in specimens. RNA pull-down assay was used for analysis of the correlation between HOTAIR and DNMT1.

**Results:**

The expression levels of the HOTAIR were up-regulated in COPD patient lungs and CSE-induced HPVEC. HPVEC apoptosis with down-regulated Bcl-2 expression, increased promoter methylation, DNMT1, Bax and Cleaved-caspase 3 expression was found in emphysema mouse model and CSE-induced HPVEC. Knockdown HOTAIR can attenuate cell apoptosis and emphysema via DNMT1 mediated hypermethylation of Bcl-2 promoter in mice. In vitro, HOTAIR can aggravate the apoptosis of CSE-exposed HPVEC. DNMT1 was a target of HOTAIR and had a positive correlation with HOTAIR.

**Conclusion:**

HOTAIR facilitates HPVEC apoptosis via DNMT1 mediated hypermethylation of Bcl-2 promoter in COPD, and attenuating the expression of HOTAIR may be a new therapy to prevent COPD.

**Supplementary Information:**

The online version contains supplementary material available at 10.1186/s12931-022-02234-z.

## Introduction

Chronic obstructive pulmonary disease (COPD) is a global public health challenge due to its high prevalence and related mortality [1, 2]. China Pulmonary Health (CPH) study reported the overall prevalence of spirometry-defined COPD was 8.6% among the general Chinese population aged 20 years or older and the estimated total number of individuals was 99.9 million [3]. Cigarette smoking is a well-known risk factor for COPD and our previous research found that cigarette smoking participates in disease progression through endothelial apoptosis [4–11]. The dysregulation of DNA methyltransferase enzyme 1 (DNMT1) is considered to be the primary methyltransferase, which contributes to the de novo methylation and maintenance of DNA [12]. B-cell lymphoma-2 (Bcl-2) and the pro-apoptotic protein Bcl2-associated X protein (Bax) jointly determine whether the mitochondria will release cytochrome *c* (cyt *C*), which is the initial factor of apoptosis [13]. Our further study found that inhibition of DNA methylation can alleviate cigarette smoke extract induced (CSE-induced) endothelial apoptosis and Bcl-2 promoter methylation. Bcl-2 promoter methylation might be involved in CES-induced endothelial apoptosis [11, 14]. However, the underlying mechanisms mediating the apoptosis process remain largely unknown.

Long non-coding RNAs (LncRNAs) are functionally defined as transcripts of more than 200 nucleotides in length that have no protein-coding potential [15]. LncRNAs are being increasingly recognized to participate in many biological processes through diverse mechanisms, such as chromatin modification, protein activity regulation, gene imprinting and so on [16]. HOX transcript antisense RNA (HOTAIR) is the first lncRNA found to have a trans-acting effect. It can positively or negatively regulate cell proliferation, apoptosis, invasion and other life processes by binding to the promoter region of downstream genes [17–19].

Guo Liu’s findings identified an essential role of HOTAIR in promoting ultraviolet-induced apoptosis and inflammatory injury by up-regulating PKR in keratinocytes [17]. Another study showed that silenced HOTAIR reduced DNMT1 protein expressions in prostate cancer cells [18]. However, whether HOTAIR has a role in the progress of COPD by regulating the function of pulmonary vascular endothelial cell remains unclear.

Based on the above studies, we postulated that HOTAIR facilitates pulmonary vascular endothelial cell apoptosis via DNMT1 mediated hypermethylation of Bcl-2 promoter in COPD.

## Methods

### Lung tissue samples

This study was approved by the Clinical Trial and Ethics Committee of the Second Xiangya Hospital of Central South University and was performed in accordance with the Declaration of Helsinki. All the patients provided informed consent. The EC protocol number is: No. ChiCTR-POC-2017126. We included 13 subjects: nonsmokers (n = 4), and smokers with COPD (n = 5) and without COPD (n = 4). Peripheral lung tissue samples were obtained from subjects who underwent resection for solitary pulmonary nodule or lung cancers at the Second Xiangya Hospital of Central South University. The tissue specimens were resected at least 5 cm away from the lesion location. The diagnosis of COPD was based on the Global Initiative for Chronic Obstructive Lung Disease (GOLD) 2017 [1]. The patients with COPD had airflow limitation (forced expiratory volume in 1 s/forced vital capacity [FEV1/FVC] < 0.7). All the patients were in a clinically stable state without pulmonary infection in the last 4 weeks. Smokers were defined as subjects who had a history of at least 20 pack-years of cigarette smoking [2]. Lung tissue samples were immediately frozen in liquid nitrogen and stored at − 80 °C for further experiments, or fixed in 4% formaldehyde and cut into 3.5-mm-thick sections for immunostaining and TUNEL staining.

### Animals

This animal protocol was approved by the Ethics Committee of the Second Xiangya Hospital of Central South University. Our experiment divided the mice (C57BL/6J, 6 weeks old, male) into five groups (n = 5 each group). The control group was exposed to normal air from 8 to 16 weeks old, and the CS group was exposed to CS four times a day during the same period [10, 11]. The CS group was also treated with lenti-empty (10^8^ pfu per mouse, once a week, intratracheally) at 6 and 7 weeks old, and then exposed to CS from 8 to 20 weeks old. The last two groups were administrated lenti-HOTAIR shRNA (10^8^ pfu per mouse, once a week, intratracheally) at 6 and 7 weeks old, or lenti-HOTAIR shRNA plus Lenti-DNMT1 Expressing Vector (10^8^ pfu per mouse, once a week, intratracheally) at 7 weeks old [9]. All groups were labeled as the control, CSE, CSE + Si-HOTAIR, CSE + Vector and CSE + Si-HOTAIR + OE-DNMT1 groups. One CSE + Si-HOTAIR mouse died during the experiment, and two CSE + Si-HOTAIR + OE-DNMT1 mice died during the experiment. Consistent with the human samples, the left lung tissues of mice were inflated with 10% formalin at a constant pressure of 25 cm H_2_O for 24 h and subsequently fixed and embedded. The protein was extracted by following the same protocol as that used for the human samples.

### Cell lines and culture

Human pulmonary vascular endothelial cells (HPVECs) were purchased from the Chinese Academy of Sciences (Shanghai, China) and cultured in DMEM (Hyclone, Logan, UT, USA) supplemented with 10% fetal bovine serum and 50 U/mL penicillin and streptomycin (Gibco, Thermo Fisher Scientific, Waltham, MA, USA) at 37 °C in a 5% CO_2_ culture chamber. Starvation for 24 h was performed before exposure to CS, shRNA, and/or lentivirus. Surface labeling of HPVEC was determined by Recombinant Von Willebrand Factor (VWF) and visualized by fluorescence microscopy. HOTAIR and the vector lentivirus were transfected into HPVECs following the manufacturer’s instructions. Lentivirus was transfected into the HPVECs at a multiplicity of infection of 10. Cells positive for green fluorescent protein were considered infected.

### CSE preparation

CSE was prepared as described previously [20, 21]. Cigarettes (Furong, Changde Cigarette Company, Hunan, China) were combusted using a modified syringe-driven apparatus (products of 5 cigarettes were collected in 10 mL PBS for animal experiments, and products of 1 cigarette were collected in 20 mL Dulbecco’s minimum essential media [DMEM] for cell experiments). Each cigarette contained 12 mg tar, 1.1 mg nicotine, and 14 mg carbon monoxide. The smoke was bubbled through DMEM/PBS then filtered through a 0.2-μm pore-size filter. The 100% CSE sample was titrated to a pH of 7.2–7.4 and diluted with DMEM/PBS to obtain the required concentration. CSE was freshly prepared for every experiment.

### Morphology and apoptosis assessment

Lung tissue samples were fixed in 4% formaldehyde, cut into 3.5-mm-thick sections, and stained with hematoxylin and eosin (HE). Emphysema was quantified based on the degree of alveolar destruction, determined through measuring the MLI and DI. MLI was assessed by dividing the length of a line drawn across the section by the total number of intercepts encountered in 36 lines per sample, and 10 random fields per sample were observed by microscopy at a magnification of 100× [22, 23]. DI was assessed by dividing the number of destroyed alveoli by the total number of alveoli counted, and an average of five different sections was observed in each sample under microscopy at a magnification of 100×. Alveolar destruction alveoli was defined on the basis of the following criteria: at least 2 alveolar wall defects, at least 2 intraluminal parenchymal rags in alveolar ducts, clearly abnormal morphology, or classic emphysematous changes in the lung [24].

TUNEL staining was performed to estimate the apoptosis level in the lung tissue with an in-situ apoptosis detection kit (Shanghai Yisheng Biotech, China). The apoptotic index (AI) was determined in lung tissue from each subject to detect the apoptosis status of the lung parenchyma, and was calculated as the percentage of TUNEL-positive nuclei out of a total of more than 3000 nuclei randomly at 400× magnification. Fields containing non-parenchymal structures such as large airways or vessels were excluded [6].

### Immunoblotting

The extracted protein was separated on an SDS-PAGE gel (Beyotime, China) and transferred to a nitrocellulose (NC) membrane (Millipore, USA). Following protein transfer, the membrane was blocked with 5% nonfat milk, and washed. Then, the membrane was incubated overnight with antibodies against DNMT1 (NB100-56519, Novus Biologicals, USA), Bcl-2 (26593-1-AP, Proteintech, USA), Bax (50599-2-Ig, Proteintech, USA), cleaved-caspase3 (19677-1-AP, Proteintech, USA) and β-actin (66009-1-Ig, Proteintech, USA). After being washed four times with PBST, the membrane was incubated with HRP conjugated IgG (Jackson Immuno Research Laboratories, USA) for 1 h at room temperature. Protein band detection was performed using an ECL kit (Thermo, USA), and films were developed and fixed by a developer and fixer kit (Beyotime, China). The blots were quantitated with Quality-one software (Bio-Rad Laboratories, CA).The data were normalized to β-actin levels.

### Real time reverse transcriptase-polymerase chain reaction

RNA was extracted as previously described. RNA was reverse-transcribed using the PrimeScript RT reagent kit (Takara, China), and assayed using SYBR (Takara, China) following the manufacturer’s instructions. All of the primers were obtained from Sangon Shanghai, China (Additional file 1). Real time polymerase chain reaction (PCR) was carried out on the Step-one ABI Real-time RT-PCR system. All mRNA expression values were presented relative to β-actin.

### RNA in situ hybridization (RNA-ISH)

For cell lines, cells were placed on slides and fixed in 4% formaldehyde for 60 min, followed by protease digestion (2.5 g/mL) at 23 °C to 25 °C. The cells were then incubated in order at 40 °C with the following solutions: target probes in hybridization buffer A for 3 h; amplifier (2 nmol/L) in hybridization buffer B at 40 °C for 15 min; and label probe (2 nmol/L) in hybridization buffer C (5 SSC, 0.3% lithium dodecyl sulfate, blocking reagents) for 15 min. After each hybridization step, slides were washed with wash buffer (0.1 SSC, 0.03% lithium dodecyl sulfate) three times at room temperature [25].

### Methylation-specific PCR (MSP) assay

The Bcl-2 promoter in human was determined to range from − 3000 to − 70 bp by the Transcriptional Regulatory Element Database from Cold Spring Harbor (http://rulai.cshl.edu/cgibin/TRED/tred.cgiprocess=promInfo&pid=19717). The Bcl-2 promoter in mouse was searched in the Transcriptional Regulator Element Database (accession number 46672, NM 009741). The CpG island in the promoter (− 1867 to − 1541 bp) was detected using the UCSC Genome Browser, and the methylation status was analyzed using MSP. Primers (Additional file 1) for MSP were designed through MethPrimer (http://www.urogene.org/methprimer/), and then were blasted and confirmed using methBLAST. A Genomic DNA Extraction kit (Takara, Japan) was used to extract DNA from the lungs. The bisulfite conversion of DNA was performed with an EpiTect Bisulfite Kit (QIAGEN, Netherlands) by following the manufacturer’s instructions. Subsequently, nested PCR was performed on the bisulfate-modification samples.

### RNA pull-down assay

A total of 1 × 10^7^ HPVECs were harvested, lysed and sonicated. The HOTAIR probe was used for incubation with Streptomycin magnetic beads (Life Technologies) at 25 °C for 2 h to generate probe-coated beads. Cell lysate with HOTAIR probe or oligo probe was incubated at 4 °C for one night. After washing with wash buffer, the RNA mix bound to the beads was eluted and extracted with an RNeasy Mini Kit (QIAGEN) for RT-PCR or real-time PCR [26].

### Statistical analysis

The data were analysed using Statistical Package for Social Sciences (SPSS) version 21.0 and R software version 3.6.2 (R Foundation for Statistical Computing). The values were described as the means ± SD. One-way ANOVA and Kruskal–Wallis tests were performed to evaluate each group of data. Statistical significance was set at P < 0.05.

## Results

### Expression of HOTAIR is specifically up-regulated in COPD patient lungs and CSE-induced HPVEC

**Fig. 1:**
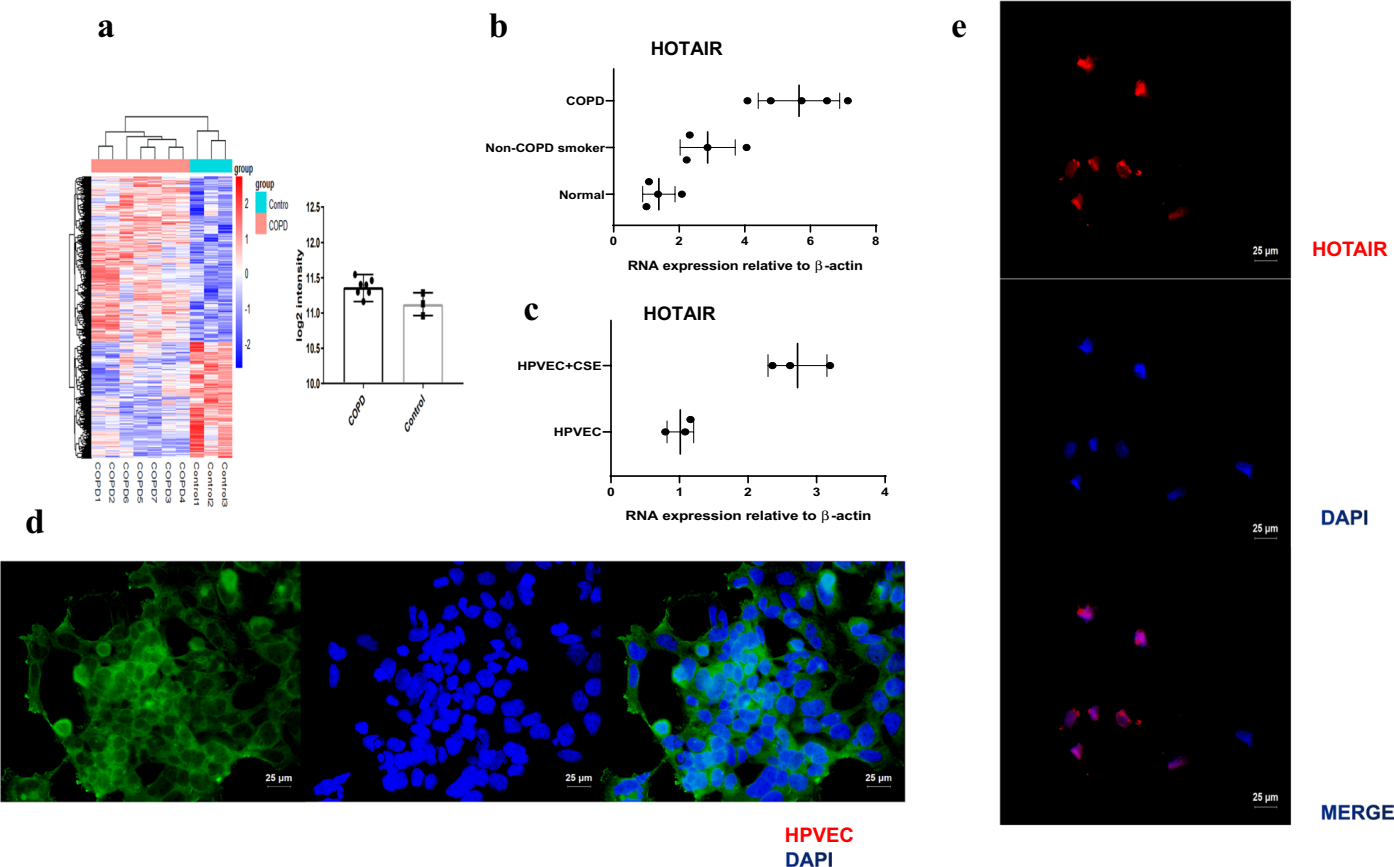
Expression of HOTAIR is specifically up-regulated in COPD patient lungs and CSE-induced HPVEC. **a** Hierarchical clustering showed the differential expression of HOTAIR in COPD (red bar) and non-COPD (blue bar) lung tissues. The expression was displayed on a scale from light (low) to deep (high). **b** Expression of HOTAIR in patient lungs from normal (nonsmokers) (n = 4), non-COPD smokers (n = 4) and COPD smokers (n = 5) was determined by qRT-PCR. β-actin was used as a reference gene. **c** Expression of HOTAIR in HPVEC (n = 3) and CSE-induced HPVEC (n = 3) was determined by qRT-PCR. β-actin was used as a reference gene. **d** Surface labeling of human pulmonary vascular endothelial cells was determined by Recombinant Von Willebrand Factor (VWF) and visualized by fluorescence microscopy. Scale bar = 25 μm. **e** Expression of HOTAIR in HPVEC was determined by RNA in situ hybridization (RNA-ISH) and visualized by fluorescence microscopy. Scale bar = 25 μm

We have collected 13 patients, who were underwent the excision of peripheral solitary pulmonary nodule or pulmonary lesion. 4 patients were normal nonsmokers, and 4 cases were non-COPD smokers. The last five cases were smokers with spirometry-defined COPD. LncRNA array was used to measure the differentially expressed lncRNAs in COPD and non-COPD lung tissues [27]. In COPD patients, HOTAIR was significantly (≥ twofold change and P < 0.05) up-regulated by Hierarchical clustering (Fig. 1a). Analysis with quantitative real-time reverse transcriptase-PCR (qRT-PCR) verified over-expression of HOTAIR in COPD smokers, whereas the expression in nonsmokers and non-COPD smokers was very low (P < 0.01) (Fig. 1b). Similarly, we detected the expression of HOTAIR in HPVEC and CSE-induced HPVEC, and labeled HPVEC with Recombinant Von Willebrand Factor (VWF) (Fig. 1d). Among these, HOTAIR was the most up-regulated lncRNA in CSE-induced HPVEC compared with HPVEC (P < 0.01) (Fig. 1c). In addition, the analysis of HOTAIR expression with RNA in situ hybridization (RNA-ISH) revealed specific mainly nuclear signal for HOTAIR in HPVEC (Fig. 1e). These results indicated that HOTAIR may have an important role in COPD.

### The location of HOTAIR is determined in COPD patient lungs by RNA-ISH

**Fig. 2:**
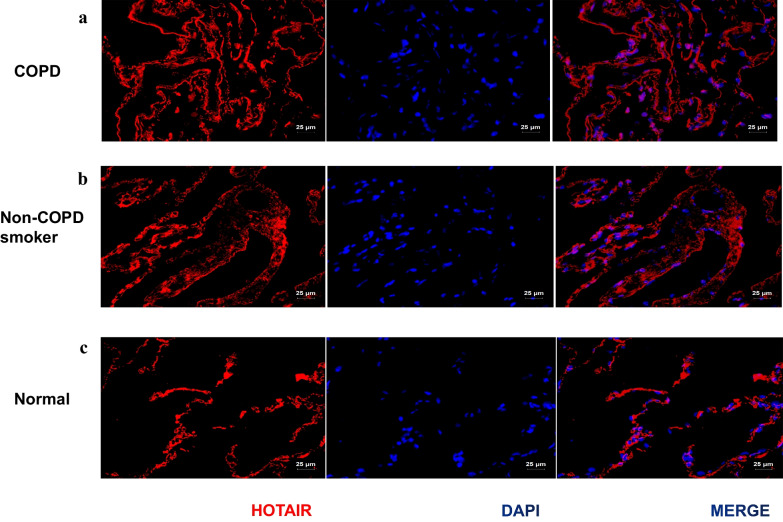
The location of HOTAIR is determined in COPD patient lungs by RNA in situ hybridization (RNA-ISH). **a**–**c** Expression of HOTAIR in paraffin-embedded tissue sections of normal (nonsmokers) (n = 4), non-COPD smokers (n = 4) and COPD smokers (n = 5). HOTAIR was analyzed with RNA-ISH and the specific location was detected in the lung tissue. Scale bar = 25 μm

To study the expression of HOTAIR during COPD progression in vivo, tissue micro-arrays consisting of tissue samples representing different stages of COPD, that is, nonsmokers, non-COPD smokers, and COPD smokers were analyzed using RNA-ISH. Expression of HOTAIR was detected in lung tissue in COPD smokers, whereas less correlated signal was detected in nonsmokers. Besides, analysis of the HOTAIR positive tissue sections revealed that it was located in the nucleus (Fig. 2a–c).

This suggested that HOTAIR may play a role in the nuclei of COPD lung tissues.

### HOTAIR facilitates CSE-induced apoptosis via DNMT1 mediated hypermethylation of Bcl-2 promoter in HPVEC

**Fig. 3:**
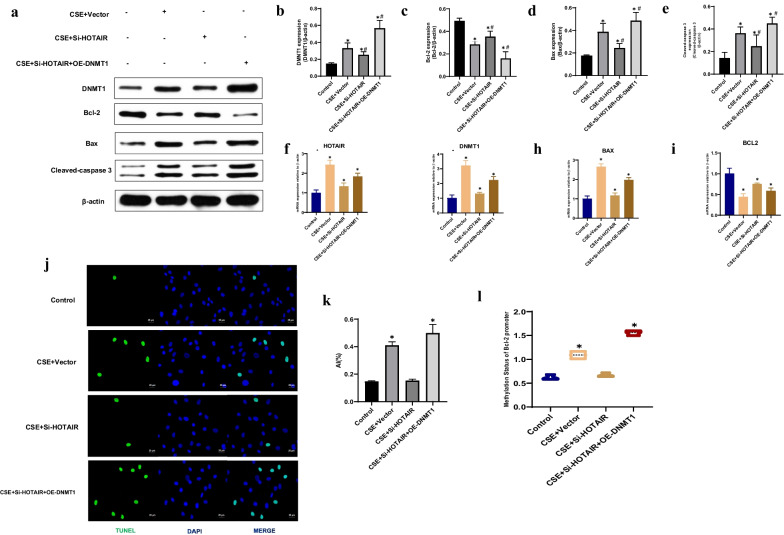
HOTAIR facilitates CSE-induced apoptosis via DNMT1 mediated hypermethylation of Bcl-2 promoter in HPVEC. **a** Immunoblotting was conducted using HPVEC from the control, CSE + Vector, CSE + Si-HOTAIR and CSE + Si-HOTAIR + OE-DNMT1 groups. **b**–**e** The relative expressions of DNMT1, Bcl-2, Bax, and Cleaved-caspase 3 in HPVEC. Results are expressed as mean ± SD. *P < 0.05 compared with the control group. ^#^P < 0.05 compared with the CSE + Vector group. **f**–**i** Expression of HOTAIR, DNMT1, Bcl-2 and Bax were measured by qRT-PCR from the control, CSE + Vector, CSE + Si-HOTAIR and CSE + Si-HOTAIR + OE-DNMT1 groups. *P < 0.05 compared with the control group. **j** TUNEL staining was conducted in HPVEC from the control, CSE + Vector, CSE + Si-HOTAIR and CSE + Si-HOTAIR + OE-DNMT1 groups. Scale bar = 25 μm. **k** Statistical analysis of the AI in different groups. Results are expressed as mean ± SD. *P < 0.05. **l** Methylation-specific PCR (MSP) was conducted using HPVEC from the control, CSE + Vector, CSE + Si-HOTAIR and CSE + Si-HOTAIR + OE-DNMT1 groups. *P < 0.05 compared with the control group

We sought to determine whether HOTAIR could facilitate the apoptosis of HPVEC exposed to CSE via DNMT1 mediated hypermethylation of Bcl-2 promoter. Immunoblotting revealed that there was higher DNMT1, Bax, Cleaved-caspase 3 and lower Bcl-2 protein levels in the CS group than in control group subjects. Compared with the CSE + vector group, the protein levels of DNMT1, Bax, and Cleaved-caspase 3 were significantly decreased in the Si-HOTAIR group, while level of Bcl-2 was significantly increased. DNMT1 over-expression in the Si-HOTAIR + OE-DNMT1 groups led to up-regulated DNMT1, Bax, and Cleaved-caspase 3 and down-regulated Bcl-2 protein levels compared with the CSE + vector group (P < 0.01) (Fig. 3a–e). Furthermore, qRT-PCR showed that there was higher HOTAIR, DNMT1, Bax and lower Bcl-2 mRNA levels in the CSE group than in control group subjects. Compared with the CSE + vector group, the mRNA levels of HOTAIR, DNMT1, and Bax were significantly decreased in the Si-HOTAIR group, while level of Bcl-2 was significantly increased. DNMT1 over-expression in the Si-HOTAIR + OE-DNMT1 groups led to up-regulated HOTAIR, DNMT1, and Bax and down-regulated Bcl-2 mRNA levels compared with the CSE + vector group (P < 0.01) (Fig. 3f–i). We next performed TUNEL assays to clarify whether increased HOTAIR was associated with apoptosis in CSE-exposed HPVEC. The results demonstrated significantly fewer TUNEL-positive cells in the control and Si-HOTAIR groups compared to the CSE and Si-HOTAIR + OE-DNMT1 groups, respectively (P < 0.01) (Fig. 3j, k). These results indicate that decreased HOTAIR could attenuate CSE-induced apoptosis in HPVEC. Given the higher expression of DNMT1 and lower expression of Bcl-2 in CSE-exposed HPVEC than in controls, we conducted Methylation-specific PCR (MSP) to detect the methylation status of the Bcl-2 promoter. The sequence results demonstrated that the CSE and Si-HOTAIR + OE-DNMT1 groups had an elevated level of Bcl-2 promoter methylation. As the results of Bcl-2 protein detection, MSP showed that there was no significant difference in Bcl-2 methylation levels between the control and Si-HOTAIR groups (Fig. 3l). Considering the simultaneously increased DNMT1 expression and methylation level, it is possible to assume that the up-regulated DNMT1 level leads to the hypermethylation of the Bcl-2 promoter in CSE-exposed HPVEC.

### HOTAIR facilitates HPVEC apoptosis via DNMT1 mediated hypermethylation of Bcl-2 promoter in the mouse model

**Fig. 4:**
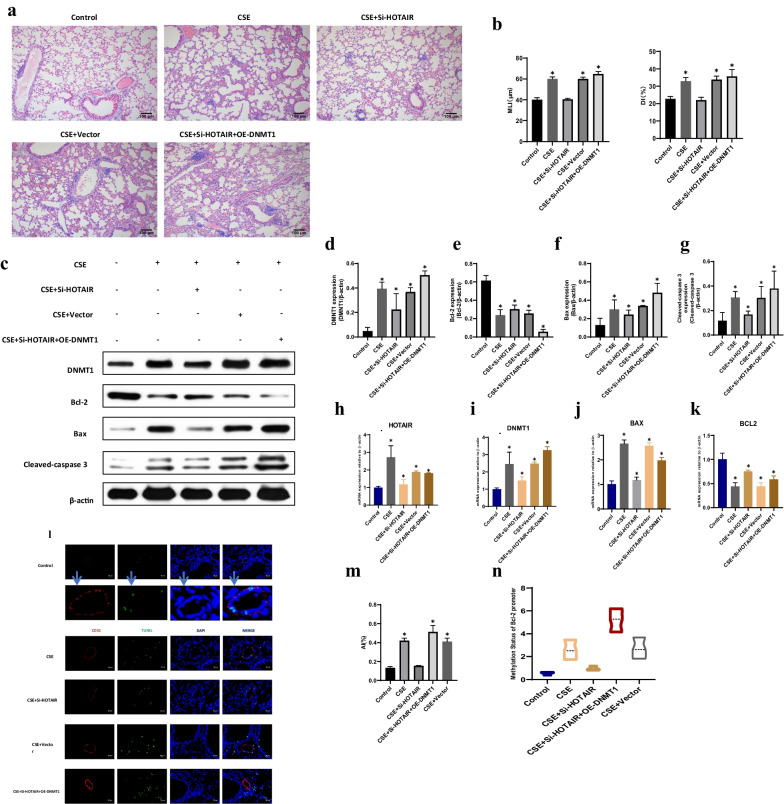
HOTAIR facilitates HPVEC apoptosis via DNMT1 mediated hypermethylation of Bcl-2 promoter in the mouse model. **a** HE staining of lungs from the control, CSE, CSE + Si-HOTAIR, CSE + Vector and CSE + Si-HOTAIR + OE-DNMT1 groups. Scale bar = 100 μm. **b** Morphometric measurements of MLI (μm) and DI (%) were performed in each group. Results are expressed as mean ± SD. *P < 0.05 compared with the control group. **c** Immunoblotting was conducted in lungs from the control, CSE, CSE + Si-HOTAIR, CSE + Vector and CSE + Si-HOTAIR + OE-DNMT1 groups. **d**–**g** The relative expressions of DNMT1, Bcl-2, Bax, and Cleaved-caspase 3 in lungs. Results are expressed as mean ± SD. *P < 0.05 compared with the control group. **h**–**k** Expression of HOTAIR, DNMT1, Bcl-2 and Bax were measured by qRT-PCR from the control, CSE, CSE + Si-HOTAIR, CSE + Vector and CSE + Si-HOTAIR + OE-DNMT1 groups. *P < 0.05 compared with the control group. **l** TUNEL staining was conducted in HPVEC from the control, CSE, CSE + Si-HOTAIR, CSE + Vector and CSE + Si-HOTAIR + OE-DNMT1 groups. Scale bar = 25 μm. **m** Statistical analysis of the AI in different groups. Results are expressed as mean ± SD. *P < 0.05. **n** Methylation-specific PCR (MSP) was conducted using HPVEC from the control, CSE, CSE + Si-HOTAIR, CSE + Vector and CSE + Si-HOTAIR + OE-DNMT1 groups. *P < 0.05 compared with the control group

Because of the elevated HOTAIR mRNA level and hypermethylation of Bcl-2 in the lung tissue of emphysema subjects, we tested whether modulation of the HOTAIR mRNA level or activity ameliorates emphysema, pulmonary apoptosis and Bcl-2 promoter hypermethylation in mouse models. The mice were depleted of HOTAIR intratracheally using a lentiviral delivery system [10^8^ plaque-forming units (pfu) per mouse] and subsequently exposed to CS. In mice exposed to CS, the MLI and DI values were significantly increased in the CSE group compared with the control group, and the values were decreased in Si-HOTAIR group compared with CSE + vector and Si-HOTAIR + OE-DNMT1 groups. Moreover, CS-treated mice exhibited emphysematous changes with aggravated MLI and DI (P < 0.01) (Fig. 4a, b). Similarly, Immunoblotting revealed that there was higher Bcl-2 and lower DNMT1, Bax, Cleaved-caspase 3 protein levels in Si-HOTAIR group than in the CSE + vector and Si-HOTAIR + OE-DNMT1 group subjects (P < 0.01) (Fig. 4c–g). Notably, qRT-PCR showed that the mRNA levels of HOTAIR, DNMT1, and Bax were significantly decreased in the Si-HOTAIR group, while level of Bcl-2 was significantly increased compared with the CSE + Vector and Si-HOTAIR + OE-DNMT1 groups (P < 0.01) (Fig. 4h–k). TUNEL staining also showed less pulmonary apoptosis in the Si-HOTAIR group than in the CSE + Vector and Si-HOTAIR + OE-DNMT1 groups (P < 0.01) (Fig. 4l–m). Furthermore, HOTAIR knockdown mice presented decreased methylation levels of the Bcl-2 promoter in the lungs (P < 0.01) (Fig. 4n). This result indicates that HOTAIR gene silencing prevented emphysema, pulmonary apoptosis, downregulated Bcl-2 expression, increased Bax, Cleaved-caspase 3 levels and Bcl-2 promoter hypermethylation in CS exposed mice.

### HOTAIR is required for DNMT1 mediated hypermethylation of Bcl-2 promoter

**Fig. 5:**
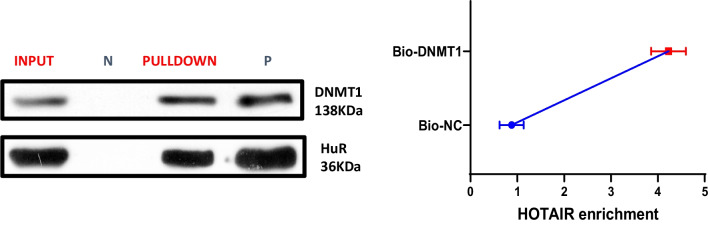
HOTAIR is required for DNMT1 mediated hypermethylation of Bcl-2 promoter. The targeting relations of HOTAIR and DNMT1 were confirmed by RNA pull-down assay. Endogenous HOTAIR was enriched specifically in DNMT1 probe detection compared with control group

Bioinformatics prediction showed that lncRNA HOTAIR could potentially bind to DNMT1. QRT-PCR assay also showed that DNMT1 expressions were significantly lower than control group after lncRNA HOTAIR knockdown. Thus, we then explored the relationship of HOTAIR and DNMT1. We employed biotinylated DNMT1 probe to pull down the lncRNA HOTAIR. Data indicated endogenous lncRNA HOTAIR was enriched specifically in DNMT1 detection compared with control group, suggesting that DNMT1 is a direct promotion target of lncRNA HOTAIR (P < 0.01) (Fig. 5).

## Discussion

Our results showed that HOTAIR mRNA levels were significantly up-regulated in the HPVEC of patients with COPD, and HOTAIR down-expression can attenuate CSE-induced cell apoptosis and emphysema via DNMT1 mediated hypermethylation of Bcl-2 promoter in mice. In vitro, HOTAIR can aggravate the apoptosis of CSE-exposed HPVEC. Moreover, HOTAIR targeted DNMT1 and promoted its expression. These findings illustrate that HOTAIR facilitates HPVEC apoptosis via DNMT1 mediated hypermethylation of Bcl-2 promoter in COPD.

LncRNAs have been shown to be essential regulators of transcription and translation through their interactions with DNAs, RNAs, and proteins over the past few decades [28–30]. HOTAIR is one of the important lncRNAs in various tumor carcinogenesis and highly expressed in various cancers, which is closely related to tumor size, advanced and extensive metastasis [26, 31–33]. However, the role that HOTAIR plays in COPD pathogenesis remains to be determined. To address this problem, we used LncRNA array analysis to study the differential expression of lncRNAs in COPD and non-COPD lung tissues in the previous study. Three hundred lncRNAs including HOTAIR were upregulated in COPD lung tissues compared to non-COPD tissues [27]. These results suggested that there were significant changes in lncRNA profile in COPD pathological processes. We found HOTAIR was significantly up-regulated by Hierarchical clustering in COPD patients, but it was not possible to identify the specific transcript. Then we searched for HOTAIR at NCBI Reference Sequences and Nucleotide database, and there was no mention of which transcript was “predominant”. Therefore, when there is no transcript requirement, it is generally designed for the whole gene (the default is the homologous region of all transcripts), without any specific transcript. So the result is representative of the commonality that all transcripts exhibit. Our study also disclosed that HOTAIR acted as a crucial regulator in COPD development and facilitated HPVEC apoptosis and promoted DNMT1-hypermethylation in vitro and in vivo.

Bcl-2 is a widely accepted anti-apoptotic regulator that maintains the mitochondrial membrane and controls the activation of the caspase family [34]. It was demonstrated that decreased Bcl-2/Bax caused apoptosis, by releasing cytosolic cyt C from mitochondria [35]. Consistent with previous studies [9, 11], our results revealed that Bcl-2 expression was significantly lower in CSE-induced HPVEC and CS-exposed lung tissues in mice than in control groups. HOTAIR knockdown mice presented increased levels of Bcl-2 and decreased levels of Bax and Cleaved-caspase 3 in the lungs, which indicates that HOTAIR gene silencing prevented emphysema and pulmonary apoptosis in CS exposed mice. Promoter methylation is an emerging and essential pre-transcriptional regulation mechanism that attach methyl groups to cytosine bases adjacent to guanine (CpG sites). There is a CpG island in the promoter of Bcl-2 in both human and mouse, which is rich in CpG sites and has the potential of methylation [36]. Interestingly, we found that there was no significant difference in Bcl-2 methylation levels between the control and Si-HOTAIR groups. Considering the simultaneously increased HOTAIR expression and methylation level, it is possible to assume that methylation initiation and inhibition are closely related to HOTAIR.

Some previous researches demonstrated that miRNAs can modulate LncRNA HOTAIR expression, including miR-141, miR-148a, miR-34a and miR-20a-5p [26, 37–39]. To further clarify the relationship of HOTAIR and DNMT1, we employed biotinylated DNMT1 probe to pull down the lncRNA HOTAIR. The consequence indicated endogenous lncRNA HOTAIR was enriched specifically in DNMT1 detection, suggesting that DNMT1 is a direct promotion target of HOTAIR. In this study, we investigated the epigenetic mechanism of HOTAIR in COPD, and found that HOTAIR promoted the methylation of Bcl-2 by upregulating DNMT1. DNA methylation is mediated by DNMT1, DNMT3a and DNMT3b, which catalyze cytosine-C5 methylation in the context of CpG dinucleotide. DNMT1 is known to be responsible for maintaining DNA methylation [40]. Several recent studies have found that LncRNA modulated the stability of DNMT1, leading to the DNA methylation of tumor suppressor genes [41]. They also revealed that abnormal methylation of tumor suppressor genes have been associated with clinicopathological features and clinical outcomes in cancers, whereas the promoter methylation appears to be a relatively early event during cancers [42]. Consequently, targeting DNMT1 could be a potential target in the treatment of COPD.

However, there are some limitations that exist in our study. First, the limited samples might not fully confirm the accuracy of the results.

Secondly, we did not conduct detailed investigation of genes that comprise the LncRNA HOTAIR/miRNAs axis after we determined that DNMT1 is a direct promotion target of lncRNA HOTAIR. We should also yield further insight into the mechanism by which lncRNA HOTAIR overexpression induces COPD progression. And the relationship between HOTAIR and other potential targeting miRNAs needed more attentions and researches. Thirdly, our previous studies [6, 43] found that not only endothelial cells, but also epithelial cells were involved in emphysema. Whether there is an interaction between epithelial and endothelial cells is attractive and complicated. Therefore, the cellular mechanism will be discussed in our further study.

## Conclusion

In summary, LncRNA HOTAIR mRNA levels were up-regulated significantly in the HPVEC of patients with COPD. HOTAIR can aggravate CSE-induced cell apoptosis and emphysema in mice and HPVEC. Moreover, HOTAIR targeted DNMT1 and promoted its expression. These findings demonstrate that HOTAIR facilitates HPVEC apoptosis via DNMT1 mediated hypermethylation of Bcl-2 promoter in COPD, and attenuating the expression of HOTAIR may be a new therapy to prevent COPD.

## Supplementary Information

**Additional file 1.** Primers for real time-PCR and MSP.

## Data Availability

The datasets used and/or analysed during the current study available from the corresponding author on reasonable request.

## Abbreviations

COPD: Chronic obstructive pulmonary disease
HPVEC: Pulmonary vascular endothelial cell
CSE: Cigarette smoke extract
RNA-ISH: RNA in situ hybridization
DNMT1: DNA methyltransferase enzyme 1
Bcl-2: B-cell lymphoma-2
Bax: Bcl-2-associated X protein
TUNEL: Terminal deoxynucleotidyl transferase dUTP nick end labeling
MSP: Methylation-specific PCR
LncRNAs: Long non-coding RNAs
HOTAIR: HOX transcript antisense RNA
GOLD: Global Initiative for Chronic Obstructive Lung Disease
VWF: Recombinant Von Willebrand Factor
HE: Hematoxylin and eosin
AI: Apoptotic index
