# Supplementary material for: Long noncoding RNA HOTAIR facilitates pulmonary vascular endothelial cell apoptosis via DNMT1 mediated hypermethylation of Bcl-2 promoter in COPD
Source: Respir Res. 2022 Dec 17;23:356. doi: 10.1186/s12931-022-02234-z (PMC9758792; doi:10.1186/s12931-022-02234-z)
Supplement: Supplementary file 1 — Additional file 1. Primers for real time-PCR and MSP. [file 12931_2022_2234_MOESM1_ESM.docx]

Supplementary material

1 All of the primers of Real time reverse transcriptase-polymerase chain reaction

.M- beta actin

<http://www.ncbi.nlm.nih.gov/gene/11461>

F ACATCCGTAAAGACCTCTATGCC

R TACTCCTGCTTGCTGATCCAC

Product length 223bp

M-BAX

<https://www.ncbi.nlm.nih.gov/gene/12028>

F TGAAGACAGGGGCCTTTTTG

R AATTCGCCGGAGACACTCG

product length 140bp

M-DNMT1

<https://www.ncbi.nlm.nih.gov/gene/13433>

F AAGCAGAACAAGGACCGCATC

R CAGTCGCCCACCTCTAGCAT

Product length 113bp

M-HOTAIR

<https://www.ncbi.nlm.nih.gov/gene/100503872>

F GCACATCTATCTCCACCGCA

R CCACAGGTTCCGTGTGTGTA

Product length 124bp

M-Bcl-2

<https://www.ncbi.nlm.nih.gov/gene/12367>

F TCTGACTGGAAAGCCGAAACTCT

R AGCCATCTCCTCATCAGTCCCA

Product length 100bp

2 All of the primers of Methylation-specific PCR (MSP) assay

M Pair

M-F GTTATTTAGGTTAGCGGTCGCGGT

M-R CCTAATTTCCTATACGTACGTCACCGTT

Product length 170bp

U Pair

M-F GTTATTTAGGTTAGTGGTTGTGGT

M-R CCTAATTTCCTATACATACATCACCATT

Product length 170bp

3 The image of agarose gel with RT- sample.


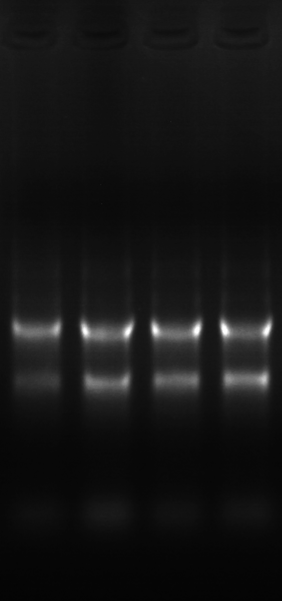


4 RNA-ISH. The results of negative controls.


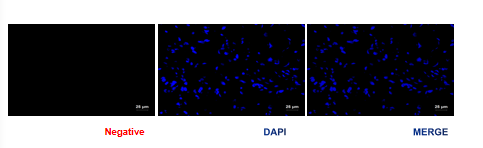


5 The sequences of shRNAs.

Product number Name The target sequence

siB12221153433 si-h-HOTAIR_007 CCACATGAACGCCCAGAGA

siB12523160128 si-h-HOTAIR_001 CCACGAAGCTAGAGAGAGA

siB1211292216 si-h-HOTAIR_004 GAGCTTGATCCGAAAGCTT

siB161213030518 si-m-Hotair_001 CCACTTTGCTGCTGTGGAA

siB161213030535 si-m-Hotair_002 GCCTATTAGAATCCAGGAT

siB161213030551 si-m-Hotair_003 CCAGGTTCAGGCCTTACTT

6 The catalog and batch numbers of each kit used.

Kit Numbers

In-situ apoptosis detection kit Shanghai Yisheng 40306ES50

RNA-ISH Ruibo C10910

7 The amount of antibody used for each assay.

Antibody Amount and dilution ratio

BAX 1:5000

Bcl2 1:1000

Cleaved-caspase3 1:1000

DNMT1 0.25μg/ml

Actin 1:5000

HRP goat anti-mouse IgG 1:5000

HRP goat anti-Rabbit IgG 1:6000

CD31 1:50

HuR 1：1000
